# Diversity of Cnidarian Muscles: Function, Anatomy, Development and Regeneration

**DOI:** 10.3389/fcell.2016.00157

**Published:** 2017-01-23

**Authors:** Lucas Leclère, Eric Röttinger

**Affiliations:** ^1^Sorbonne Universités, UPMC Univ Paris 06, CNRS, Laboratoire de Biologie du Développement de Villefranche-sur-mer (LBDV)Villefranche-sur-mer, France; ^2^Université Côte d'Azur, CNRS, INSERM, Institute for Research on Cancer and Aging (IRCAN)Nice, France

**Keywords:** cnidaria, muscle, myoepithelial cells, development, regeneration, evolution, epitheliomuscular cells

## Abstract

The ability to perform muscle contractions is one of the most important and distinctive features of eumetazoans. As the sister group to bilaterians, cnidarians (sea anemones, corals, jellyfish, and hydroids) hold an informative phylogenetic position for understanding muscle evolution. Here, we review current knowledge on muscle function, diversity, development, regeneration and evolution in cnidarians. Cnidarian muscles are involved in various activities, such as feeding, escape, locomotion and defense, in close association with the nervous system. This variety is reflected in the large diversity of muscle organizations found in Cnidaria. Smooth epithelial muscle is thought to be the most common type, and is inferred to be the ancestral muscle type for Cnidaria, while striated muscle fibers and non-epithelial myocytes would have been convergently acquired within Cnidaria. Current knowledge of cnidarian muscle development and its regeneration is limited. While orthologs of myogenic regulatory factors such as MyoD have yet to be found in cnidarian genomes, striated muscle formation potentially involves well-conserved myogenic genes, such as *twist* and *mef2*. Although satellite cells have yet to be identified in cnidarians, muscle plasticity (e.g., de- and re-differentiation, fiber repolarization) in a regenerative context and its potential role during regeneration has started to be addressed in a few cnidarian systems. The development of novel tools to study those organisms has created new opportunities to investigate in depth the development and regeneration of cnidarian muscle cells and how they contribute to the regenerative process.

## Introduction

Muscles, tissues specialized for contraction, are an essential component of the eumetazoan (all animals except sponges and placozoans) body. They are involved in various functions of the body and are well characterized in various vertebrate and main non-vertebrate models (reviewed in Schmidt-Rhaesa, 2007; Bryson-Richardson and Currie, 2008; Bentzinger et al., 2012; Andrikou and Arnone, 2015; Almada and Wagers, 2016). In bilaterians, muscles are rich in myofilaments (organized arrays composed principally of actin and myosin II) and present two basic types of cells: true muscle cells (myocytes) and myoepithelial cells. Myocytes are individual muscle cells, usually not anchored to the extracellular matrix (ECM), which during embryogenesis derive mainly (but not exclusively) from the mesoderm layer. In contrast, myoepithelial cells, which have a variety of embryological origins, are anchored to the ECM and are fully integrated into an epithelial tissue layer. Both of these muscle cell-types can be further defined as either striated or smooth, depending on the internal organization of the myofilaments. Visible striations represent repeating functional units of the muscle (the sarcomeres), which result from aligned rows of alternating antiparallel actin and myosin myofilaments, spaced by their supporting Z-discs. Conversely, in smooth muscles, the myofilaments are organized irregularly.

The diversity of muscle organizations is best characterized in mammals. There are four muscular organizations: two are striated, named skeletal and cardiac muscles; the other two are the smooth and myoepithelial muscles (Alberts et al., 2015). In skeletal muscles, myocytes fuse to form multinucleated syncytia called muscle fibers or myotubes. In contrast, cardiac and smooth muscles are composed of mononucleated muscle cells for which mechanical, chemical, and electrical coupling is possible via complex junctions (adherens and gap), forming the typical “intercalated disc” structures of cardiac muscles. Myoepithelial cells in mammals are generally found in glandular epithelia such as the mammary or salivary glands and display a double identity, smooth muscle and epithelial cell (Petersen and van Deurs, 1989). In non-vertebrate bilaterians, striated and smooth myocytes as well as myoepithelial muscles are also present (reviewed in Schmidt-Rhaesa, 2007). Smooth and striated muscle cells can either be mono- or multinucleated, as described for example in *Drosophila* (Susic-Jung et al., 2012). Although myogenesis, muscle physiology and muscle regeneration have been extensively described and studied in bilaterians (reviewed in Bryson-Richardson and Currie, 2008; Bentzinger et al., 2012; Andrikou and Arnone, 2015; Almada and Wagers, 2016), less is known about their evolutionary origin(s) (Dayraud et al., 2012; Steinmetz et al., 2012; Brunet et al., 2016) as well as function, development and plasticity of muscles in non-bilaterian animals.

**Figure 1:**
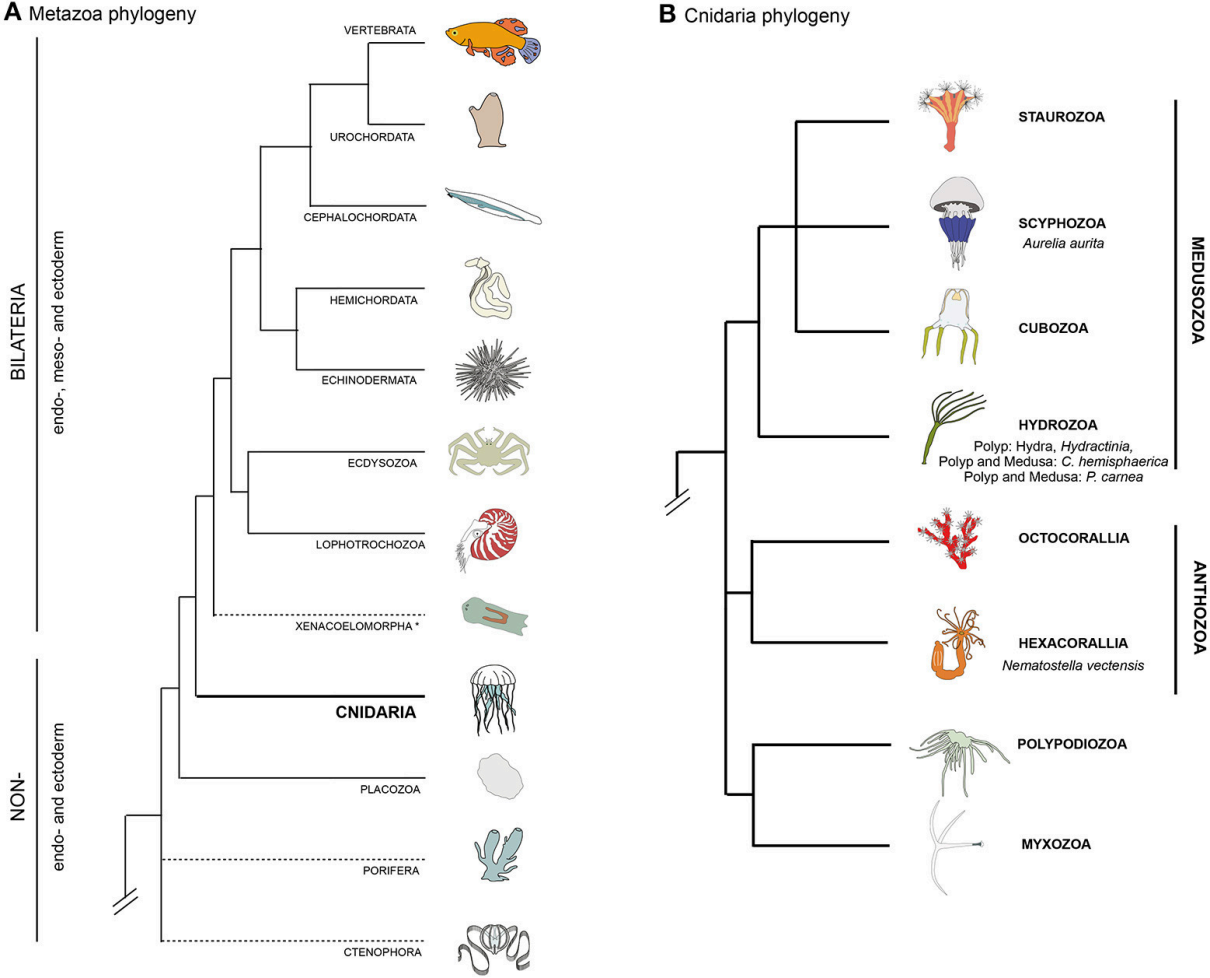
**Bilaterian and cnidarian phyolgenies. (A)** Metazoan phylogeny, highlighting the pivotal position of cnidarians as the sister group to extant bilaterian animals. The position of Ctenophora and Porifera (sponges) outside the Bilateria remains controversial (as indicated by dashed lines). **(B)** Cnidarian phylogeny showing the relationships between the main lineages based on recently published data (Chang et al., 2015; Zapata et al., 2015).

A group of organisms that has attracted a long standing interest in this research area is the Cnidaria (Chapman et al., 1962; Quaglia, 1981; Seipel and Schmid, 2005, 2006). This group of animals, as the sister group to the bilaterian clade (Figure 1A, Chang et al., 2015; Zapata et al., 2015), holds a key phylogenetic position for understanding muscle evolution. The two main groups of the phylum Cnidaria are Anthozoa and Medusozoa (Figure 1B). Anthozoa (sea anemones, corals) are mostly sessile and are represented by individual or colony-forming polyps arising from the metamorphosis of a planula larva. Medusozoa (jellyfish, hydroids) form in some species a free-swimming medusa (jellyfish), in addition to the polyp and planula stages. Beside anthozoans and medusozoans, a group of parasites, myxozoans, have recently been formally identified as cnidarians on the basis of molecular phylogenies (Figure 1B) (Chang et al., 2015) and presence of cnidarian specific genes (Holland et al., 2011; Shpirer et al., 2014). They have been proposed to be the sister group to another cnidarian parasitic species, *Polypodium hydriforme* (Chang et al., 2015), forming the clade Endocnidozoa (Zrzavý and Hypša, 2003).

A handful of cnidarians has emerged in the past decades as experimental models in molecular, cell and developmental biology, providing insights into the evolution of developmental programs, including regeneration, stem cell biology and the evolution of key bilaterian traits (Kraus et al., 2007, 2016; Momose and Houliston, 2007; Amiel et al., 2009; Chera et al., 2009; Boehm et al., 2012; Layden et al., 2012; Röttinger et al., 2012; Sinigaglia et al., 2013; Leclère and Rentzsch, 2014; Abrams et al., 2015; Bradshaw et al., 2015; Helm et al., 2015; reviewed in Technau and Steele, 2011; Layden et al., 2016; Leclère et al., 2016; Rentzsch and Technau, 2016). The main, but not exclusive, cnidarian models are the medusozoan hydrozoans *Hydra, Hydractinia, Podocoryna* and *Clytia* (reviewed in Houliston et al., 2010; Galliot, 2012; Plickert et al., 2012; Gahan et al., 2016; Leclère et al., 2016) as well as the anthozoans *Nematostella vectensis* (reviewed in Layden et al., 2016; Rentzsch and Technau, 2016) and the coral *Acropora* (Shinzato et al., 2011; Hayward et al., 2015; Okubo et al., 2016).

**Figure 2:**
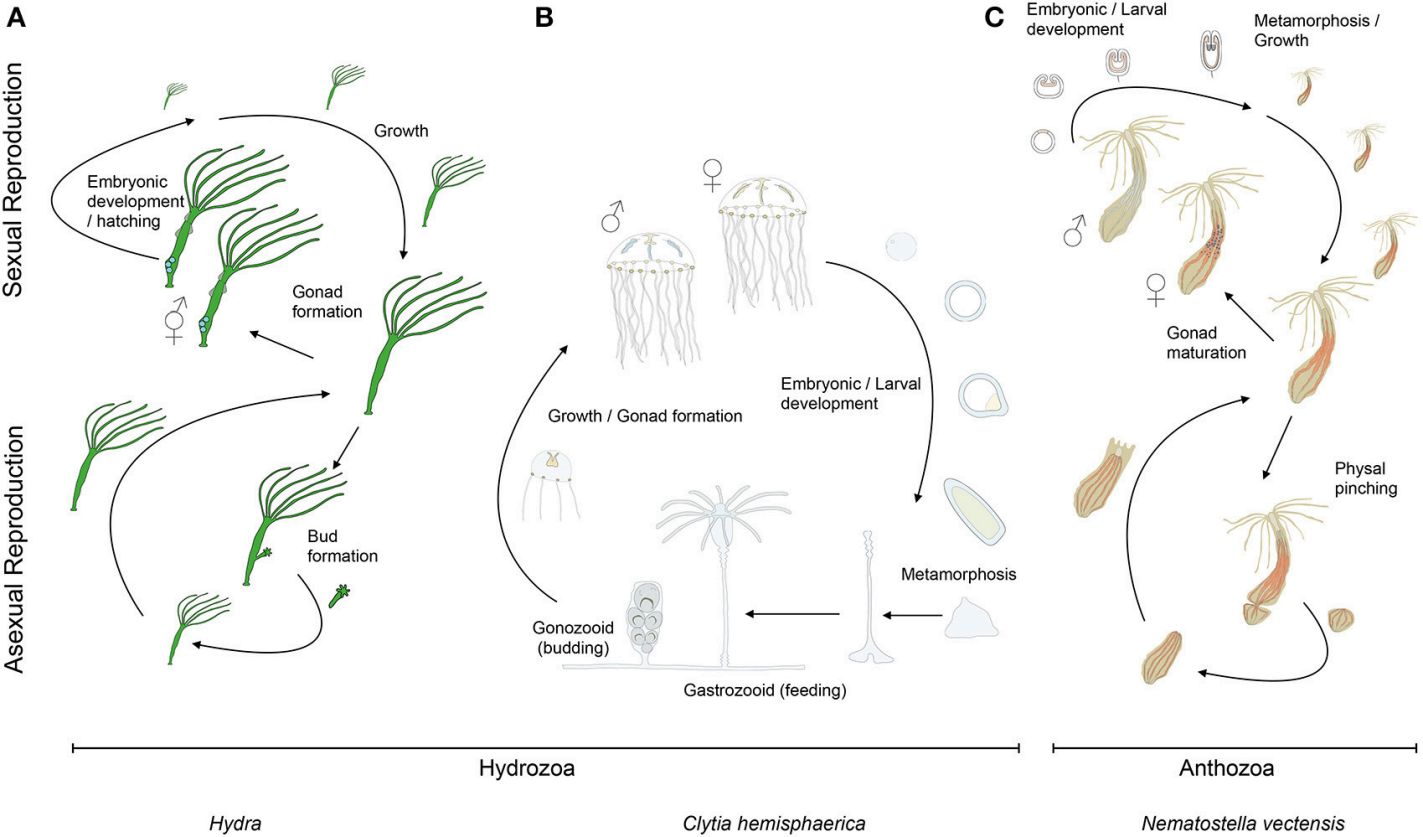
**Cnidarian life cycles**. The life cycles of **(A)** the solitary fresh water polyp *Hydra*, **(B)** the marine jellyfish *Clytia* (both hydrozoans) and **(C)** the anthozoan polyp *Nematostella*. At the lower part of the panels are indicated their asexual reproductive potentials (budding, physal pinching) that give rise to new **(A)** *Hydra* or **(C)** *Nematostella* polyps, or **(B)** juvenile *Clytia* medusae, respectively. Under harsh environmental conditions, gonads develop and sexual reproduction in **(A)** *Hydra* can occur. Depending on the species, *Hydra* can be gonochoric or hermaphroditic (represented here). After fertilization, embryonic development occurs within a solid capsule that, after hatching, frees a juvenile *Hydra*. **(B)** *Clytia* and **(C)** *Nematostella* are gonochoric and oocytes and sperm are released into the water column. After fertilization, embryonic development leads to the formation of swimming planula larvae that after metamorphosis develop into **(B)** a polyp colony for *Clytia* or **(C)** a solitary juvenile polyp for *Nematostella*.

Cnidarians display a broad variety of muscle organizations performing various functions. Unlike bilaterians, the main muscle cell type of cnidarians is the epitheliomuscular cell, a specialized epithelial cell containing smooth myofilaments, and which constitutes the principal building block of the two body layers (ectodermal and endodermal epithelia, also referred as epidermis and gastrodermis for both polyps and medusae, e.g., Brusca and Brusca, 2003; Schmidt-Rhaesa, 2007). The terms “epitheliomuscular cell” and “myoepithelial cell” are often used interchangeably (e.g., Brusca and Brusca, 2003). Some authors, however, apply morphology-based definitions: “epitheliomuscular cells” are exposed to both sides of the epithelium, while “myoepithelial cells” have reduced apical ends and are not exposed to the apical surface (e.g., Ruppert et al., 2004). Following most of the literature, here we simply define those terms taxonomically, using “epitheliomuscular cells” and “myoepithelial cells” when referring to the myofilaments-containing epithelial cells of, respectively, cnidarians and bilaterians. Other muscle types are also found in Cnidaria, such as the striated muscle of the medusa required for swimming. The complex life cycles and high regenerative capabilities found in Cnidaria involve a remarkable plasticity of muscle systems, which can take on different configurations during the life cycle of a given species (Figure 2). Cnidaria display both similarities and differences to its sister group, the bilaterians, with respect to muscle organization and cellular constituents. Additional data from cnidarian muscles can therefore provide important insights into their ontogeny, function and plasticity, in particular within an evolutionary framework. In this review, we discuss muscle diversity, function, development and regeneration in cnidarians. We conclude by proposing that cnidarians, in addition to increasing our understanding of metazoan muscle evolution, may also provide new insights into the development/regeneration and (re-) patterning of epitheliomuscular/myoepithelial cells, as well as into the role that muscle fibers play in the regeneration process.

## Cnidarian muscle functions

Cnidarian muscles play crucial roles in locomotion, defense from predators (e.g., contracting and burying in crevices/sand), feeding and digestion through continuous peristaltic movements (Shimizu et al., 2004, Figure 3). In the following section we briefly review the described functions of muscles at each stage of the cnidarian life cycle and the known connections to the nervous system.

**Figure 3:**
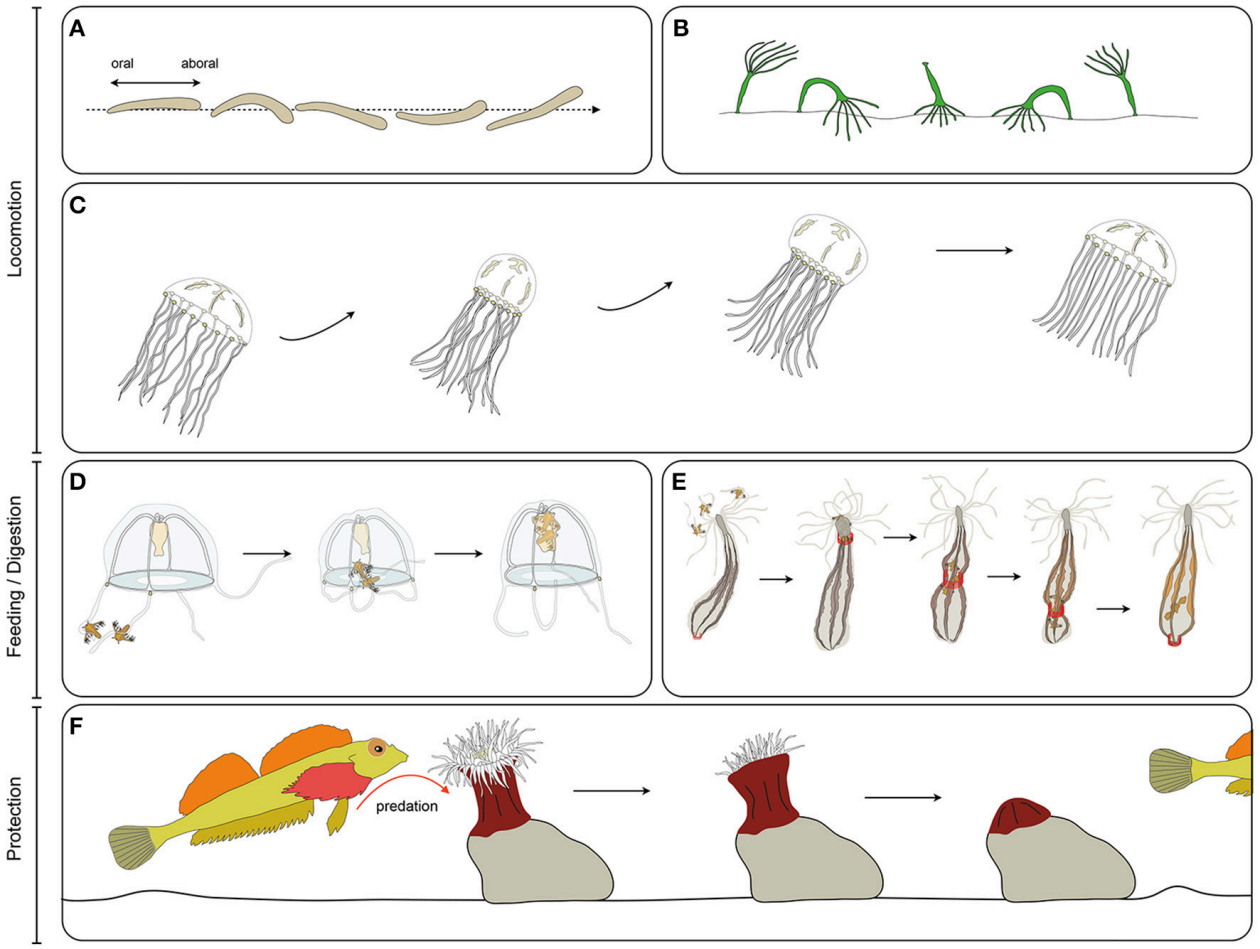
**Cnidarian muscle functions. (A)** Planula larva crawling, **(B)**, *Hydra* polyp somersaulting, **(C)** jellyfish pulsation, **(D)** guided tentacle retraction of the jellyfish to bring the food toward the mouth, **(E)** digestive peristaltic movements of the polyp (red rings along the body column indicate circumferential muscle contractions), **(F)** protective retraction of the polyp in response to predation pressure.

Most of cnidarian muscle cells are epitheliomuscular and one distinctive feature of those cells compared to muscles cells of other animal groups is their multifunctionality. In *Hydra* for instance, endodermal epitheliomuscular cells participate in nutrient absorption during the digestion process (Buzgariu et al., 2015). Epitheliomuscular cells in the ectoderm of the foot produce vesicles containing an adhesive substance responsible for attachment to the substrate, while specialized epitheliomuscular ectodermal cells, the “battery cells,” function as supporting cells for the nematocytes (Hufnagel et al., 1985; Campbell, 1987). In many anthozoans, epitheliomuscular cells of the endodermal body wall host a large population of dinoflagellate symbionts, and take part in the digestive process, mixing the content of the gastrovascular cavity via beating of apical cilia and performing intracellular digestion (Hyman, 1940). Multifunctionality is thought to be an ancestral characteristic of epitheliomuscular/myoepithelial cells (Arendt, 2008). The inherent multifunctional potential of epitheliomuscular cells has been recently demonstrated in *Hydra*, whose epitheliomuscular cells displayed a remarkable functional plasticity (Wenger et al., 2016). The authors showed that in strains lacking nerve cells, the expression of several neurogenesis- and neurotransmission-specific genes was upregulated in epitheliomuscular cells, thus suggesting they could compensate for the loss of the nervous system by extending their multifunctionality.

### Muscle functions at the planula stage

At the planula stage, movement is mostly mediated by cilia beating of the ectodermal cells. However, in a number of species, bending along the oral-aboral axis to modulate the swimming direction is muscle dependent, such as in the hydrozoan *Clava multicornis*, in which muscles allow bending of the larvae for efficient phototaxis (Figure 3A; Piraino et al., 2011). How this coordinated behavior is regulated by the nervous system of the planula larva is not yet known.

### Muscle functions at the polyp stage

At the polyp stage, muscle contraction drives a wide variety of behavior: rhythmic contraction, mouth opening (Passano and McCullough, 1963; Carter et al., 2016), prey capture and handling (Miglietta and Tommasa, 2000), contracting or extending tentacle in order to regulate oxygen, waste and symbiont exposure (Bell et al., 2006), defense, escape (Figure 3F) and protection by retraction (Miglietta and Tommasa, 2000; Swain et al., 2015), peristaltic movements allowing fluid circulation within the body cavity and facilitating digestion (Figure 3E; Anctil et al., 2005), and locomotion. *Hydra* is notably able to move via a complex array of movements, including rare instances of somersaulting (alternative attachment and release of the foot and tentacles combined with contraction and extension of the body column, Figure 3B) (Trembley, 1744; Ewer and Fox, 1947). Many sea anemones (Actiniaria) are able to perform creeping using the muscles of their pedal disk (McClendon, 1906), their tentacles (Ross and Sutton, 1961) or to burrow using peristaltic movements (Williams, 2003). Some sea anemones are able to swim through sharp flexions of the column (Yentsch and Pierce, 1955; Ross and Sutton, 1967) or synchronous lashing of the tentacles (Josephson and March, 1966; Robson, 1966).

Polyps contract and extend efficiently even though their muscles are not organized in pairs of antagonists as in many bilaterian animals. In many cases, extension and retraction movements are performed by perpendicularly oriented muscles, as for example in the *Hydra* polyps: longitudinal ectodermal muscles are involved in contraction while endodermal circular muscles are involved in polyp extension.

All the above mentioned behaviors are regulated by the nervous system (reviewed in Galliot et al., 2009). Some involve rhythmic contraction of the body column. In *Hydra*, a pacemaker system regulates this process (Passano and McCullough, 1963, 1964; Kass-Simon et al., 2003; Ruggieri et al., 2004). It is constituted by a small subset of nerve cells connected by gap junctions located near the foot, and capable of synchronous firing (Takaku et al., 2014). Comparable pacemaker systems have been described in other cnidarian polyps, such as the hydrozoan *Tubularia* (Josephson and Uhrich, 1969; de Kruijf, 1977) and the swimming anthozoan sea anemone *Stomphia* (Robson, 1961, 1963). Chemical synapses between nerve cells and epitheliomuscular cells have been shown to be widespread in Cnidaria (Westfall et al., 1971; Westfall, 1973). They presumably contain neuropeptides and at least several components of the bilaterian neuromuscular junctions (Chapman et al., 2010).

### Muscle functions at the medusa stage

Medusae inhabit the water column, and move by means of passive drifting in the water currents, combined with active swimming. Muscles are not only used for propulsion, but also for many other functions such as catching prey, bringing food to the mouth (often showing a great amount of coordination with the movements of the bell and of the manubrium—stalk-like structure bearing the mouth – Figure 3D), digestion and dispersion of gametes (see for e.g., Passano, 1973; Bourmaud and Gravier-Bonnet, 2004). Rhythmic contraction and extension of the medusae bell are mediated differently; contraction of the medusa bell is the result of contraction of the striated muscle of the subumbrella, while viscoelastic (Alexander, 1964) and elastic properties (Demont and Gosline, 1988) of the medusa mesoglea (the thick layer of extracellular matrix located between the two epithelia) counteract muscle contraction and allow the bell to regain its original shape at each contraction cycle (Figure 3C).

Swimming efficiency has been studied in several species, and has been shown to depend on several parameters affecting hydrodynamics, such as the overall shape of the medusa, the disposition of the muscles in the subumbrella, the flexibility of the bell margin and the shape of the velum (bi-layered epithelium running around the rim of the bell, present in hydrozoan medusae, and reducing the size of the bell cavity opening) (Dabiri et al., 2005, 2010; Colin et al., 2012). Rhythmic contractions of the bell has been shown to be a very efficient process for underwater propulsion (Gemmell et al., 2013). A recent study recreated this configuration artificially using chemically dissociated rat heart muscle laid on ephyra (juvenile scyphozoan medusa) shaped silicone polymers (Nawroth et al., 2012). This demonstrated that the rhythmic contraction of the rat muscle cells activated by a periodic electrical stimulation, coupled to the elasticity of the polymer, is sufficient to generate efficient propulsion of the artificial jellyfish.

Even if medusae are generally considered to be “simple” organisms, coordination of muscle-based locomotion and integration of spatial information can be quite complex, at least in some species. For instance, several cubomedusae display courtship behaviors (Lewis and Long, 2005), perform obstacle avoidance (Garm et al., 2007) or even use terrestrial visual cues for navigation through mangroves forests (Garm et al., 2011). Similarly, the scyphomedusa *Rhizostoma pulmo* has recently been shown to actively swim countercurrent in response to current drift (Fossette et al., 2015). Directionality and propulsion can also result from the coordination of multiple individuals, as seen for instance in colonial siphonophores. In *Nanomia bijuga*, clonal medusoid individuals, termed nectophores, propel the colony, and developmental differences between them generate a division of labor that ultimately modulates locomotion (Costello et al., 2015).

Jellyfish contractions are regulated by a complex nervous system, including neural nets and concentrations of nerve cells at the bell margin called the nerve rings (reviewed in Satterlie, 2011). Pacemaker neurons regulating bell margin contractions have been described in cubozoan, scyphozoan and hydrozoan jellyfish (reviewed in Satterlie and Nolen, 2001; Mackie, 2004; Katsuki and Greenspan, 2013). How the photoreceptor systems control the swim pacemaker has started to be addressed in cubomedusae (Garm and Mori, 2009; Stöckl et al., 2011; Bielecki et al., 2013). In hydrozoan medusae, the contraction of striated muscle in the subumbrella is notably regulated by gap junctions, which electrically couple the muscle cells (Satterlie, 2008). This process has yet not been reported in other cnidarian groups. Finally, anatomical specialization of the nerve nets can allow for a fine tuning of movements: for example, in *Aglantha*, a complex and well-characterized neuromuscular system allows the jellyfish to swim either slowly or fast, thanks to different neural circuitries, modulating the escape response (reviewed in Mackie, 2004).

## Cnidarian muscle types

The main, and in many species exclusive, muscle cell type in cnidarians is the epitheliomuscular cell. These cells have a typical polarized epithelial morphology, including apical cilia, with the specificity that myofibrils project from the basal side, aligning within the extracellular matrix of the tissue to provide its contractile property. In a few cnidarians, smooth muscles are found totally embedded in the mesoglea, having lost contact with the epithelia (see below for more details). Interestingly though, in most of the free-swimming medusae, muscles are composed of striated epitheliomusclar cells. Despite the few muscle cell types found in cnidarians, there is a wide diversity of muscle systems in this phylum. In this section we describe briefly the diversity of muscle organization and muscle cell types described in the major groups of cnidarians.

### Muscle systems in hydrozoans

**Figure 4:**
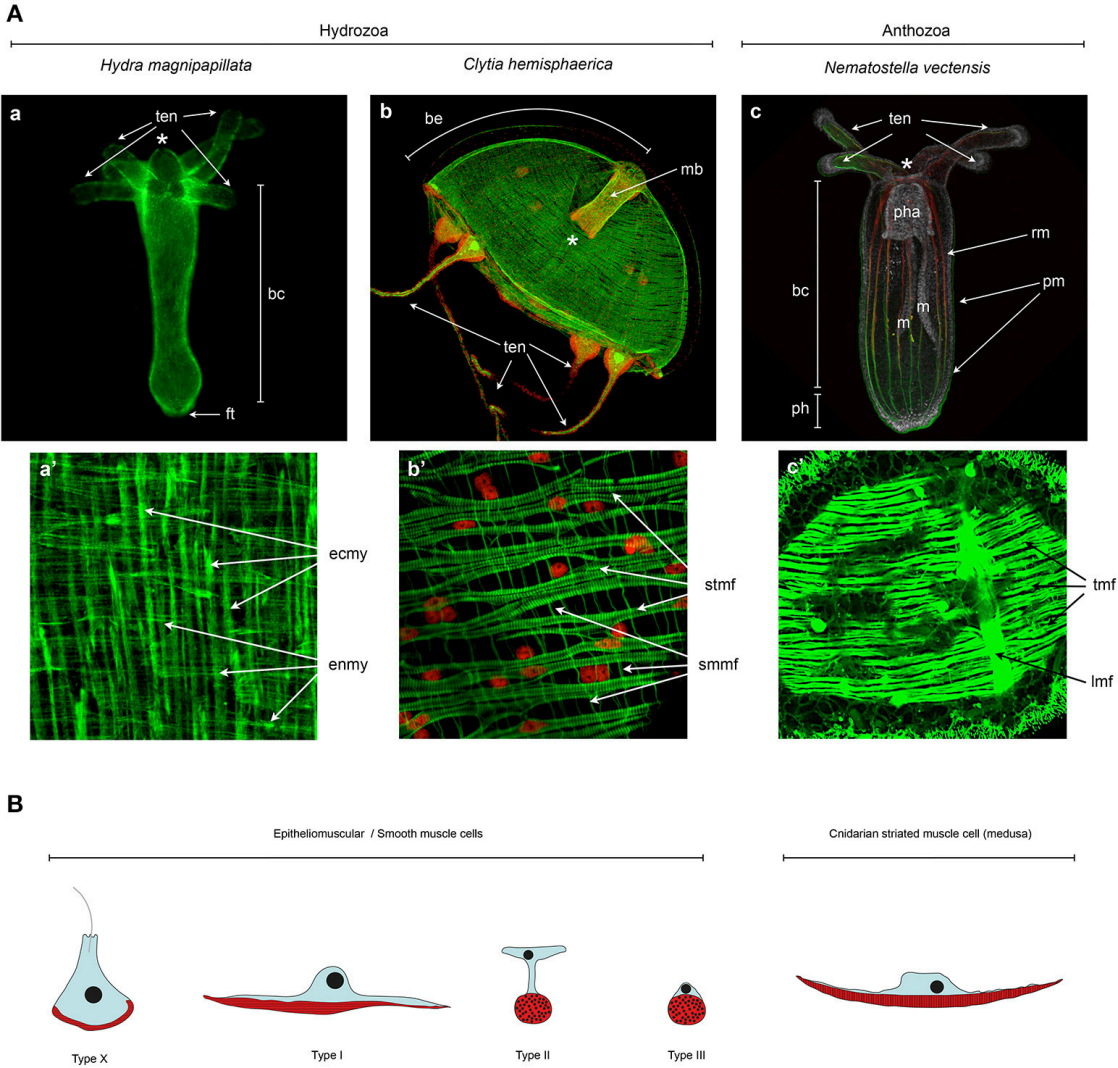
**Cnidarian muscle diversity. (A)** Muscle networks of **(a,a')** *Hydra magnipapillata*, **(b,b')** *Clytia hemisphaerica* jellyfish and **(c,c')** *Nematostella vectensis*. The upper panels show the muscle network of entire organisms and the lower panel magnification of certain body regions to highlight the orientation and fine structure of the muscles. **(A)** Living Lifeact:GFP transgene (Seybold et al., 2016) labeling actin filaments, **(c)** fixed MyHC1::mCherry (Renfer et al., 2010) transgene labeling the actin fibers of the retractor muscles, co-labeled with phalloidin. All other panels **(a',b,b',c')** are phalloidin stainings. Image labels are as follows: (^*^) mouth, (ten) tentacles, (bc) body column, (ft) foot, (be) bell, (mb) manubrium, (pha) pharynx, (m) mesentery, (ph) physa, (rm) retractor muscle, (pm) parietal muscle, (ecmy) ectodermal myonemes, (enmy) endodermal myonemes, (stmf) striated muscle fibers, (smmf) smooth-like muscle fibers, (tmf) transversal muscle fibers, (lmf) loongitudinal muscle fibers. **(a,a')** Image courtesy of Aufschnaiter and Hobmayer, **(b,b')** images from Kraus et al. (2015) and **(c,c')** Image from Amiel et al. (2015) as well as courtesy of Amiel. **(B)** Epitheliomuscular cell type diversity in Cnidaria. After Krasińska (1914) and Doumenc (1979) in Seipel and Schmid (2006), and Jahnel et al. (2014).

Most ectodermal and endodermal epithelial cells in hydrozoan planulae, polyps, and medusae are epitheliomuscular (West, 1978). Much of the available information about hydrozoan epitheliomuscular cells comes from anatomical and physiological studies on *Hydra* polyps. *Hydra* ectodermal and endodermal epitheliomuscular cells display, respectively, longitudinally and circularly oriented processes, called myonemes (Figures 4Aa,a'; Mueller, 1950). Ectodermal epitheliomuscular cells of *Hydra* are large columnar or cuboidal cells bearing two long myonemes oriented along the oral-aboral axis (David, 1973). These two myonemes of roughly cylindrical shape, composed of irregularly arranged myofilaments, are found in each ectodermal cell, as visualized by electron microscopy (West, 1978) or recently by LifeAct-GFP transgenic polyps (Seybold et al., 2016). Instead, endodermal epitheliomuscular cells are tall and columnar, have short muscle processes at the basal end and several flagella at the apical end. Their myonemes are oriented perpendicularly and have a structure similar to those found in the ectoderm, though more numerous (David, 1973; Seybold et al., 2016). Epitheliomuscular cells of the body column (both in the endoderm and ectoderm) divide continuously, thus displacing cells toward the oral (mouth) and aboral (foot) extremities where they are ultimately eliminated (Campbell, 1967).

Each epitheliomuscular cell process of a *Hydra* polyp is in contact with the basal processes of several adjacent cells, thus forming a continuous muscle fiber network spanning the entire body (Mueller, 1950). It should be noted here that the term “muscle fiber” is generally associated to the multinucleated syncytia of skeletal muscles; following most of the literature on cnidarian muscle, we will use henceforth this term to indicate condensed actin filaments constituting the contractile elements of cnidarian muscles. Adjacent epitheliomuscular cells in *Hydra* are connected by septate and gap junctions; additionally, where the myoneme-containing regions of two adjacent cells come into contact, they form a characteristic and unique type of junction, which structurally resembles the intercalated discs found in vertebrate cardiac muscles: on the inner surface of each cell membrane is an irregular band of dense material through which the filaments of the myoneme itself pass (Haynes et al., 1968).

As a general rule, the muscle fibers of hydrozoan planula larvae and polyps are circularly arranged in the endoderm and longitudinally in the ectoderm (Hyman, 1940; Bouillon, 1993). Common parts of the polyp colony also harbor epitheliomuscular cells, such as the endodermal epitheliomuscular cells of the stolon in *Podocoryna carnea* (Buss et al., 2013). However, not all hydrozoan epithelial cells are epitheliomuscular. For instance, endodermal epithelial cells of the tentacles of many hydrozoan species are arranged in only one row of turgescent cells and do not contain myofilaments (Bouillon, 1993).

The main muscle of the hydrozoan medusae is the circular striated muscle found in the subumbrella (the inner layer of the bell—Figures 4Ab,b'), responsible for the rhythmic contraction of the bell, and composed of epitheliomuscular cells. As for smooth epithelial muscles, basally located striated myofilaments are connected between neighboring cells, forming a continuous circular muscle. Each epitheliomuscular cell contains about 30–50 sarcomeres, as for instance in the hydrozoan medusae *Aglantha digitale* (Singla, 1978). Sarcomeres in a relaxed state are approximately 1 μm long. As described in various hydrozoan species (e.g., Keough and Summers, 1976; Boelsterli, 1977; Singla, 1978), they are of very similar structure compared to those of vertebrate striated muscles, being separated by Z-discs and composed of ordered arrays of thick and thin filament areas forming denser A-bands and rarer I-bands. As in vertebrates, A-bands contain a central H-band and a M-line. An interesting deviation can be observed in *Obelia* medusae (Chapman, 1968), probably linked to their unusually flat shape. In these species, the striated myofilaments of the subumbrella are not oriented circularly but distributed in two perpendicularly oriented sets, generating a grid-like pattern. In addition to the subumbrella, in most hydrozoan medusae striated epitheliomuscular cells also constitute a contractile ring on the inner layer of the velum.

In hydrozoan medusae, while swimming is generally performed by the circular striated muscles, other behaviors are mostly mediated by the smooth muscles. Hydrozoan medusae are therefore rich in smooth epitheliomuscular cells (Bouillon, 1993) such as (i) the longitudinal muscle fibers of the tentacle ectoderm, (ii) the outer layer of the velum, and (iii) the radially oriented smooth muscle fibers of the subumbrella (the underside of the bell) that run from the manubrium to the bell margin and cover the striated muscle layer. In a few species, additional epitheliomuscular cells have been described that form (iv) the ring muscle fibers of the endodermal (gastrovascular) canal system and (v) the radiating muscle fibers of the exumbrella (the outer layer of the bell).

The anatomical details of medusa muscle systems differ greatly among species, an interesting case for evo-devo studies, still to be explored. Most strikingly, the organization of the smooth muscles covering the striated muscle of the subumbrella, responsible for the bending of the medusa, shows great variation: in many species, such as *Clytia hemisphaerica*, this layer covers the entire subumbrella, while in others, such as *Podocoryna carnea*, these radial smooth muscles are organized in four bundles, each covering one of the four radial canals (Seipel and Schmid, 2006).

### Muscle systems in other medusozoans

One of the most striking feature of the muscle systems of Scyphozoa, Cubozoa, and Staurozoa compared to Hydrozoa, is the quasi-absence of muscle fibers in the endoderm at the planula, polyp and medusa stages. Past (Hyman, 1940; Chapman, 1965; Chapman and Werner, 1972; Werner et al., 1976; Anderson and Schwab, 1981; Martin and Chia, 1982; Chia et al., 1984) and more recent studies (Chapman, 1999; Eggers and Jarms, 2007; Nakanishi et al., 2008) do not report any endodermal muscle fibers. However, Gold et al. (2015) convincingly describe poorly developed circular muscle fibers in the endoderm of the polyp tentacles of *Aurelia*. Smooth and striated myofilaments found in the ectoderm, are nevertheless structurally similar to those found in Hydrozoa (Anderson and Schwab, 1981; Chia et al., 1984). As in Hydrozoa, medusa stages in Scyphozoa and Cubozoa contains strong circular epitheliomuscular striated muscles lining the subumbrella (Hyman, 1940; Satterlie et al., 2005; Helm et al., 2015), often called the coronal muscles. Most medusae of these groups also contain radial smooth muscles lining parts of the subumbrella and longitudinal ectodermal epitheliomuscular smooth muscles in the tentacles (Hyman, 1940; Satterlie et al., 2005).

Polyp stages in Scyphozoa, Cubozoa, and Staurozoa have strong longitudinal muscles, of ectodermal origin. In many species, these muscles are constituted by myocytes completely embedded in the mesoglea, and thus not connected either to the ectoderm or the endoderm epithelia (Widersten, 1966; Werner et al., 1976; Chapman, 1978; Chia et al., 1984; Westlake and Page, 2016). In addition, smooth epitheliomuscular cells are present in the ectoderm of the polyp tentacles (Franc, 1993). In several cubozoan and scyphozoan species (Chapman, 1978; Chia et al., 1984; Golz, 1993) some ectodermal cells at the polyp stage display striated muscle fibers of unknown origin and function, which certainly deserve more attention.

### Muscle systems in anthozoans

In Anthozoa, epitheliomuscular cells are present both in the ectoderm and the endoderm of planulae and polyps, the medusa stage being absent in this clade. Importantly, unlike in medusozoans where the major muscular components are localized in the ectoderm, muscles in anthozoans are more developed in the endoderm; most of the species possess in fact transversal (circumferential) and strong longitudinal endodermal muscles (Figures 4Ac,c').

Most anthozoan muscle cells are epitheliomuscular, containing smooth myofilaments. Loosely defined sarcomeres have been so far reported in the tentacles of only two sea anemones: *Aiptasia diaphana* and *Stomphia coccinea* (Amerongen and Peteya, 1980). True muscle cells totally embedded in the mesoglea have also been described in several anthozoans, such as the mesogleal sphincter musculature found in some Actiniaria and Zoantharia (Doumenc and Van Praët, 1987; Herberts, 1987; Swain et al., 2015).

Endodermal epitheliomuscular muscles in anthozoans can be generally classified into three types: (i) the circular musculature found throughout the body wall, (ii) the longitudinal parietal muscles positioned at the junction between the mesenteries (reproductive and digestive structures subdividing the gastric cavity into chambers) and the body wall, and (iii) the longitudinal retractor muscles located on one side of the mesenteries. In many anthozoan groups, the retractor muscles are arranged in a bilateral manner along the secondary body axis (called the directive axis), and constitute one of the landmarks of bilateral symmetry in these organisms (e.g., in *Nematostella*: Jahnel et al., 2014; Leclère and Rentzsch, 2014). Ectodermal muscles are for most anthozoans confined to the tentacles and the oral disc, except in some Ceriantharia, Antipatharia and Scleractinia that have longitudinal muscles in the body column ectoderm (Chevalier and Beauvais, 1987; Doumenc and Van Praët, 1987; Herberts, 1987; Tiffon, 1987; Van Praët et al., 1987).

Most descriptions of anthozoan muscles resulted from research on sea anemones (order Actiniaria) (reviewed in Doumenc and Van Praët, 1987). A recent description of the muscular system of the sea anemone *Nematostella vectensis* (Figures 4Ac,c') highlighted the existence of at least three different epitheliomuscular cell types (Figure 4B; Jahnel et al., 2014). Type I classical epitheliomuscular cells and type II epitheliomuscular cells, with elongated cytoplasmic bridges, constitute mainly the longitudinal component of the muscular system such as the parietal and retractor muscles (Figures 4A,B; Jahnel et al., 2014). Conversely, type III epitheliomuscular cells are basiepithelial muscle cells and are primarily encountered in the ectoderm of the tentacles. Many sea anemone species have extra sets of radial muscles: (i) ectodermal radial muscles in the oral disk, involved probably in mouth opening (Doumenc and Van Praët, 1987), (ii) radial muscles in the endodermal part of the mesenteries on the side opposite the retractor muscle (Doumenc, 1979), and (iii) radial muscles in the endoderm involved in pedal disk contraction (Doumenc and Van Praët, 1987). While ST myhc-positive cells are present in the oral disc of *Nematostella* (Renfer et al., 2010), a pedal disk is lacking and radial muscles in the endodermal part of the mesenteries have yet to be described in *Nematostella*.

### Muscle systems in endocnidozoans

The musculature of the myxozoan *Buddenbrockia plumatellae* and of *Polypodium hydriforme* has recently been investigated (Raikova et al., 2007; Gruhl and Okamura, 2012). They represent a unique case within cnidarians, possessing only smooth muscle cells localized in the mesoglea and lacking epithelial muscle cells altogether. In particular, the worm-like parasite *Buddenbrockia* possesses four longitudinal non epithelial smooth muscles (Gruhl and Okamura, 2012) while *Polypodium* has a complex array of smooth myocytes located in different parts of the body (Raikova et al., 2007). Many other myxozoans species are even more extremely specialized to their parasitic life style, not possessing any muscle cells (Hartikainen et al., 2014).

## Ontogeny of cnidarian muscles

Decades of developmental biology have taught us a great deal about striated muscle development in vertebrates and other bilaterian model systems, but little is known about smooth and myoepithelial muscles development. Similarly, studies on cnidarian muscle development have so far mainly focused on the epitheliomuscular striated muscles of the medusa, while the development of the predominant epithelial smooth muscle cell type and the myocyte type have so far been rather neglected.

Striated muscle development has been primarily studied in hydrozoan medusae. In most species, striated muscles of the subumbrella and the velum derive from the entocodon, a hydrozoan specific cell layer located in the early medusae buds, between the ectoderm and endoderm, considered by some authors to be homologous to the mesoderm of bilaterians (Boero et al., 1998; Seipel and Schmid, 2006), but see (Martindale et al., 2004; Burton, 2008) for an alternative opinion. In most hydrozoan species, this territory derives from the ectoderm (Boelsterli, 1977; Bouillon, 1993; Seipel and Schmid, 2006; Kraus et al., 2015). The work of Schmid and colleagues on the hydrozoan medusae *Podocoryna carnea* provided valuable data about cnidarian striated muscle differentiation and transdifferentiation (see below). The other medusozoan groups lack an entocodon and their striated muscles instead, differentiate from the ectoderm of the subumbrella. In fact, a recent study showed that the striated muscles of the scyphozoan *Chrysaora* are produced anew during ephyra formation (Helm et al., 2015).

Cnidarian epitheliomuscular cells reside in the ectodermal and/or endodermal epithelia. Their fate is probably specified during germ layer formation, but data are scarce, and it is still unclear what drives epithelial cells toward a epitheliomuscular fate in a given cnidarian, germ layer or body region. In a few species, epitheliomuscular cells derive from non-epithelial stem-cells, such as the interstitial stem cells (i-cells) of *Hydractinia echinata* (Müller et al., 2004; Künzel et al., 2010). I-cells are hydrozoan-specific stem-cells, capable of giving rise to multiple cell types, such as neurons, gametes, gland cells and nematocytes. It is worth noting that in *Hydra*, epitheliomuscular cells do not differentiate from i-cells, but solely from fate-restricted ectodermal and endodermal epithelial stem cells (Hobmayer et al., 2012).

## Molecular characterization of muscles in cnidarians

### Myogenic genes

The development of vertebrate skeletal muscles is fairly well characterized at the molecular level, while a few factors involved in vertebrate smooth muscle formation have been identified, such as Myocardin, SRF and Capsulin (a paralog of MyoR; Kumar and Owens, 2003; Wang et al., 2003). In contrast, little is known about the cellular and molecular characteristics of myoepithelial cell precursors as well as the mechanisms controlling the double “myo” and “epithelial” phenotype (Tamgadge et al., 2013).

A set of bHLH (basic helix-loop-helix) domain containing transcription factors, the Myogenic Regulatory Factors (MRFs), play key roles in vertebrate skeletal myoblast specification and differentiation. MRFs are notably able, when overexpressed, to transform fibroblasts into myoblasts (Davis et al., 1987). They are also present in non-vertebrate bilaterians where they similarly regulate specification and differentiation of striated muscles (reviewed in Andrikou and Arnone, 2015). The four vertebrate MRF paralogs—*Myf5, MyoD, Mrf4*, and *Myogenin*—resulted from vertebrate specific duplications; therefore, only one MRF ortholog, usually called MyoD, is found in most non-vertebrate bilaterian groups.

**Figure 5:**
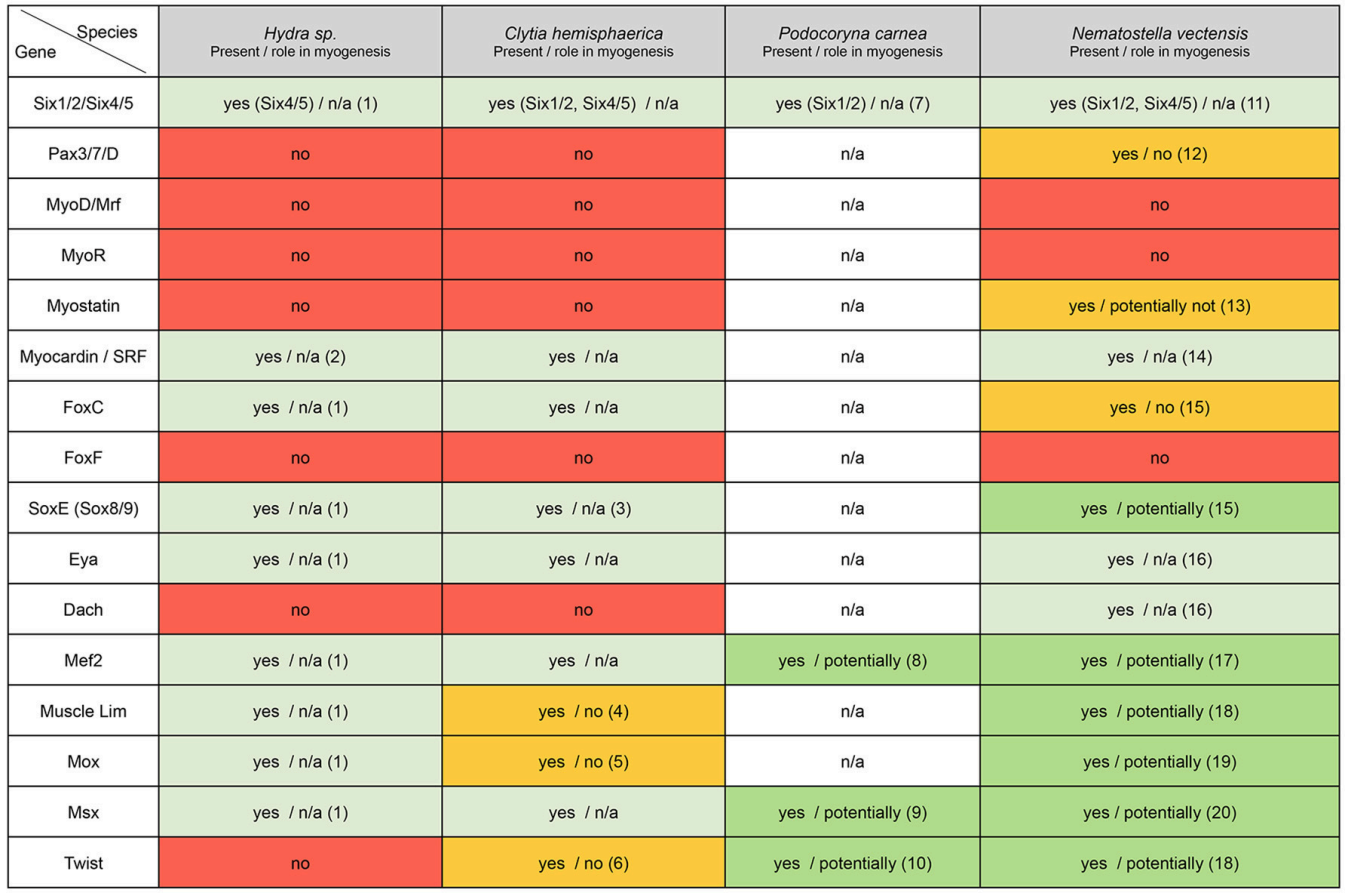
**Cnidarian “muscle” gene repertoire**. Overview of the cnidarian “muscle” gene repertoire in regard to known bilaterian myogenic factors. Cnidarians are represented by *Hydra, Clytia, Podocoryna*, and *Nematostella*. The potential role in myogenesis of a given gene in the indicated species has been assessed by functional studies if available or by published gene expression patterns, (n/a) no information available. References cited in this figure: (1) Chapman et al., 2010; (2) Hoffmann and Kroiher, 2001; (3) Jager et al., 2011; (4) Steinmetz et al., 2012; (5) Chiori et al., 2009; (6) Kraus et al., 2015; (7) Stierwald et al., 2004; (8) Spring et al., 2002; (9) Galle et al., 2005; (10) Spring et al., 2000; (11) Ryan et al., 2006; (12) Matus et al., 2007; (13) Saina and Technau, 2009; (14) Putnam et al., 2007; (15) Magie et al., 2005; (16) Nakanishi et al., 2015; (17) Genikhovich and Technau, 2011; (18) Martindale et al., 2004; (19) Ryan et al., 2007; (20) Ryan et al., 2006.

MRFs are part of a conserved myogenesis gene regulatory network, which includes the transcription factors Dach (Dachshund), Pax3, Pax7, Six1, Six4, as well as their co-factors Eya1 and Eya2 (Grifone et al., 2005; Christensen et al., 2008). MRFs are also able to induce differential transcription of specific *mef2* splice variants, a MADS family transcription factor (Potthoff and Olson, 2007; Potthoff et al., 2007). While Mef2 governs expression of a set of downstream factors including Myocardin, a protein required for muscle development (Wang et al., 2001), Mef2 *per se* does not have myogenic activity, but cooperates transcriptionally to potentiate the effects of MRFs (Molkentin et al., 1995). Two other bHLH factors, MyoR (Myogenic Repressor) and Twist negatively regulate skeletal muscle differentiation by repressing MyoD activity (Spicer et al., 1996; Hebrok et al., 1997; Lu et al., 1999). A non-exhaustive list of the major bilaterian myogenic factors is shown in Figure 5 (reviewed in Bentzinger et al., 2012; Andrikou and Arnone, 2015). The set of Pax, bHLH, Six, Eya, Dach, and MADS transcription factors involved in myogenesis is conserved throughout Bilateria. However, the hierarchy of gene interactions has been reshuffled in some bilaterian groups, and some key myogenic factors were lost in some lineages during evolution, such as Pax3/7 in sea urchins (Andrikou et al., 2015). The general consensus is that MRFs play a crucial role in bilaterian muscle specification and differentiation (reviewed in Bentzinger et al., 2012; Andrikou and Arnone, 2015).

No MRFs have been identified in the published cnidarian genomes (Putnam et al., 2007; Chapman et al., 2010; Shinzato et al., 2011), while several orthologs to other bilaterian transcription factors and signaling components related to myogenesis were found (Figure 5). Thorough phylogenetic analyses showed that a previously reported *MyoD* putative ortholog from *Podocoryna* named *JellyD1* (Müller et al., 2003), is indeed not related to the *MyoD* family of bHLH factors (Simionato et al., 2007). The absence of MRF orthologs in cnidarians raises the pivotal question of the developmental mechanisms underlying muscle formation in these organisms. An unbiased systematic analysis of genes regulating muscle formation would be particularly helpful.

The first extensive search for myogenic genes in cnidarians was carried out in the hydrozoan medusae *Podocoryna*. Volker Schmid and collaborators identified and characterized the bHLH transcription factor *Twist*, the MADS factor *mef2*, as well as the homeobox transcription factor *msx*, and showed that all three genes are expressed in the entocodon of the medusa bud and its derivatives, from which the smooth-like and striated muscles of the bell originate (Spring et al., 2000, 2002; Galle et al., 2005). While *msx* expression is downregulated in bilaterian striated muscles, striated muscles of the medusa maintain elevated levels of *msx* expression (Galle et al., 2005). The transcription factors *twist* and *mef2* are also expressed in non-muscle tissues, thus suggesting they could play additional roles during jellyfish development (Spring et al., 2000, 2002).

In the sea anemone *Nematostella*, orthologs for nearly all of the main “myogenic genes” [Figure 5, with the exception of *foxF* (Santagata et al., 2012), *myoR* and *myoD*] have been identified. Among those genes potentially involved in muscle formation, only *mef2* has been studied functionally (Genikhovich and Technau, 2011). Genikhovich and colleagues described several differentially expressed splice variants, and in particular one responsible for proper endoderm formation. Through a combination of TEM analysis and transgenic approaches, using a Myosin Heavy Chain promoter-driven mCherry [MyHC1::mCherry (Renfer et al., 2010) also called ST myhc::mCherry (Steinmetz et al., 2012)], the authors showed that longitudinal muscle formation is impaired in some *NvMef2* splice-specific morphants (Genikhovich and Technau, 2011). However, given that the defects in endoderm formation appeared prior to the condensation of actin filaments that will form the retractor muscles, and also that direct binding of NvMef2 to the ST myhc promoter is not required for the expression of the myosin reporter, the direct role of *NvMef2* is still unclear (Genikhovich and Technau, 2011). Therefore, the function of all potential myogenic factors during muscle specification and formation in cnidarians remains to be determined.

### Structural muscle genes

The essential contractile machinery—alternation of actin thin filaments and Myosin II thick filaments—is conserved between cnidarians and bilaterians. However, contrary to bilaterians, actin paralogs specific for muscle and cytoplasm have not been reported from cnidarians (Fisher and Bode, 1989). ST myhc (“striated muscle” type II Myosin Heavy Chain) is present in the thick filaments of both smooth (Renfer et al., 2010; Steinmetz et al., 2012) and striated muscles (Schuchert et al., 1993; Aerne et al., 1996; Steinmetz et al., 2012) in several cnidarians, while NM myhc (“non-muscle” type II Myosin Heavy Chain) is expressed in either smooth-muscle and non-muscle cells in *Clytia* and *Nematostella* (Steinmetz et al., 2012). This situation resembles the arrangement found in most bilaterians for which ST myhc is used in fast contracting muscles, while NM myhc functions in slow contracting muscles (Brunet et al., 2016) and constitutes an important component of the cytoskeleton (Vicente-Manzanares et al., 2009).

Several actin or myosin regulators and binding partners characterizing bilaterian muscles (reviewed in Hooper and Thuma, 2005) were also found in cnidarians. Myosin Essential and Regulatory Light Chains, Myosin Light Chain-Kinase and Phosphatase, as well as the smooth muscle ATPase regulator Calponin are present in cnidarians genomes (Steinmetz et al., 2012) but have not been functionally characterized yet. Several Tropomyosin paralogs have also been described in cnidarians (Baader et al., 1993; López de Haro et al., 1994; Gröger et al., 1999, 2000; Fujinoki et al., 2002; Steinmetz et al., 2012), including one specific to the striated muscle cells of *Podocoryna* (Gröger et al., 1999, 2000). However, Troponins, important components of the striated muscle thin filaments, have to date not been found in any cnidarian genomes (Steinmetz et al., 2012). Finally, all major components of the Dystroglycan complex, a protein complex involved in anchoring muscle fibers to the extracellular matrix in many bilaterians, have been identified in cnidarian genomes (Adams and Brancaccio, 2015) and await functional characterization.

Sarcomeres consist of a succession of thin and thick filaments organized in arrays by proteins complexes located at the Z-disks and M-lines. Recent work investigated the evolution of the most conserved Z-disk proteins (Steinmetz et al., 2012). The authors could show that most conserved proteins present in both vertebrate and Drosophila Z-disks, such as α-Actinin, Muscle-LIM and ZASP/LDB3, were present in cnidarians. However, in *Clytia* medusae, *in situ* hybridization signal was not detected in striated muscles for orthologs of the Z-disk proteins (Muscle-LIM and ZASP/LDB3), or showed ubiquitous expression (α-Actinin). Conversely, clear orthologs of Titin, the large protein which links Z-disk to thick filaments in bilaterians, could not be found. Most of the proteins regulating the organization of the M-line have yet to be investigated in cnidarians. Orthologs of Obscurin/UNC-89, giant proteins involved in M-line alignment in diverse bilaterians (Benian et al., 1996; Katzemich et al., 2012), have been identified in *Hydra, Clytia* and *Nematostella* (Steinmetz et al., 2012) and appear to be broadly expressed in striated, smooth, and non-muscle-cells.

## Origin and evolution of cnidarian muscles

It is generally accepted that smooth epitheliomuscular cells of cnidarians are homologous to bilaterian smooth muscles and myoepithelial cells (Steinmetz et al., 2012). Epitheliomuscular cells are found in all cnidarian species, except for some highly derived parasitic groups (see Section Cnidarian Muscle Types), and most of the molecular components of smooth muscle myofilaments are conserved between Cnidaria and Bilateria (Steinmetz et al., 2012). The current lack of functional data, however, does not allow discriminating whether the same regulatory cascade in Cnidaria and Bilateria controls smooth muscle development.

A recent study concluded that the striated muscles found in hydrozoan medusae originated independently from those found in bilaterians (Steinmetz et al., 2012). As described in the previous section, available cnidarian genomes lack key striated muscle proteins, such as the Troponins and the Z-disks component Titin while others, such as muscle-LIM and LDB3, were found to be excluded from striated muscle tissue in *Clytia* medusae. The structural convergence between hydrozoan and bilaterian sarcomeres represents an interesting and well-supported hypothesis, nevertheless awaiting confirmation from other cnidarian species. A stimulating possibility would be that striated muscles appeared during cnidarian evolution in concomitance with the acquisition of the medusa stage, and thus with the functional requirement for a fast-contracting swimming muscle. More work is therefore needed to understand the evolutionary tinkering that produced so similar phenotypes with different sets of proteins.

Smooth myocytes, muscles cells that lost connection to the epithelia, and are therefore totally embedded in the mesoglea, likely originated several times within Cnidaria. They have only been described in a few disparate instances, such as the sphincter muscle of some Anthozoa (in Actiniaria and Zoantharia), the longitudinal ectodermal muscles of scyphozoan and cubozoan polyps and staurozoans, and they represent the sole muscle type described in the parasitic groups Myxozoa and *Polypodium* (see Section Cnidarian Muscle Types). The most parsimonious interpretation for this pattern is that they represent clade-specific adaptations. Indeed, phylogenetic reconstructions of Zoantharia (Swain et al., 2015) and Actiniaria (Rodriguez et al., 2014) support several convergent acquisitions of myocytes within these groups. Furthermore, acquisition of true myocytes and loss of epitheliomuscular cells in the myxozoan *Buddenbrockia* and in *Polypodium* are likely a direct consequence of the adoption of a parasitic life style.

Several losses of either striated or smooth muscle cell types were inferred in Cnidaria, often in relation to the evolution of their complex life cycles. For instance, the multiple evolutionary losses of the medusa stage in Hydrozoa led to likewise losses of striated muscles (Leclère et al., 2009). As a consequence, *Hydra* does not develop striated muscle at any stage of its simplified life cycle (Nawrocki et al., 2012). Similarly, many myxozoan species completely lost muscle cells following extreme adaptation to the parasitic life style (Hartikainen et al., 2014). Genomic data analyses are still scarce (Chapman et al., 2010; Chang et al., 2015), though, and it remains to be determined how these losses impacted the structural and regulatory muscle genes.

## Muscle plasticity and regeneration in cnidarians

**Figure 6:**
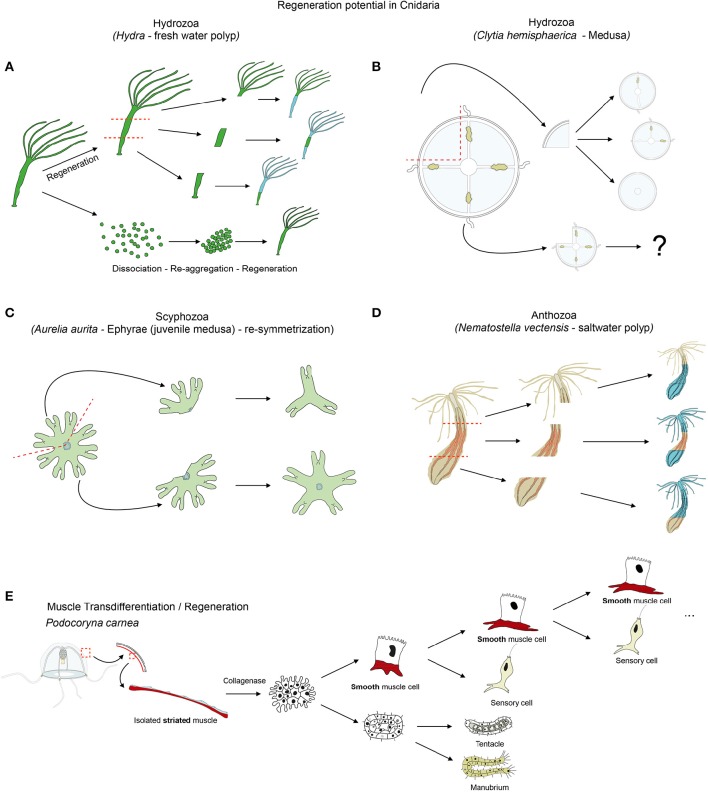
**Cnidarian regeneration potential**. Regenerative capacities of **(A)** *Hydra*, **(B)** *Clytia* medusa and **(D)** *Nematostella* as cnidarian representatives. **(C)** Illustrates the re-symmetrization process of juvenile medusa that is not regeneration *per se*, but allows a quick regain of the medusa functionality. **(E)** Illustrates the transdifferentiation and regeneration potential of striated muscle cells isolated from jellyfish and cultured *in vitro*. See text for further details.

While regeneration phenomena are widespread among metazoans, the regenerative capacity varies considerably within a given phylum and at the organ/tissue levels within an organism (Bely and Nyberg, 2010; Tiozzo and Copley, 2015). Although still quite variable within the phylum, cnidarians in general exhibit tremendous tissue plasticity and regeneration abilities (Figure 6). Our understanding about (i) muscle plasticity/muscle regeneration itself (at the tissue, cellular, and/or molecular levels), and (ii) the role that muscles play in the regenerative process of lost tissues or body parts is still sparse. In bilaterians, muscle regeneration is fueled by specific stem cells called satellite cells, however no such cells have yet been identified in cnidarians. The PaxD transcription factor Pax3/7, crucial for satellite cell activation and muscle regeneration/renewal in bilaterians (Konstantinides and Averof, 2014; reviewed in Dumont et al., 2015) has been retrieved from anthozoan genomes and further characterized in *Nematostella* (Figure 5). However, given its gene expression pattern in restricted regions of the ectoderm, it does not seem to be associated with a potential muscle renewal process (Matus et al., 2007). This rather limited set of evidence suggests that the cnidarian muscle regeneration process may differ from the one described in bilaterians. In this section we review current knowledge about muscle regeneration/plasticity in cnidarians and the potential role played by muscle cells during injury-response and remodeling processes.

### Epithelial muscle plasticity in *Hydra* polyps

A recent study on *Hydra* analyzed the repolarization of epithelial cells during the regenerative process (Seybold et al., 2016), taking advantage of the fact that dissociated *Hydra* cells are able of aggregating and regenerating a polyp. *Hydra* is indeed a classical model organism to study whole body regeneration. It can reform fully functional polyps when bisected (Trembley, 1744; Wittlieb et al., 2006), from small tissue pieces (Shimizu et al., 1993), isolated germ layers (Normandin, 1960; Kishimoto et al., 1996) and even from dissociated cell aggregates (Gierer et al., 1972; Technau et al., 2000; Seybold et al., 2016) (Figure 6A). The regenerative capacity of *Hydra* and the role of stem cells in this process have been extensively studied and reviewed elsewhere (Galliot and Schmid, 2002; Holstein et al., 2003; Bosch, 2007; Bosch et al., 2010; Galliot and Chera, 2010; Galliot and Ghila, 2010; Hobmayer et al., 2012).

Thanks to the development of a transgenic lifeact::GFP line (staining actin filaments *in vivo*, Figures 4Aa), Seybold et al. (2016) observed that polarized actin structures appeared progressively, in a cell autonomous manner, between 6 and 24 h post dissociation (hpd). Interestingly, the orientation of the reforming myonemes (in number of 2–3 per cell) appears to be polarized within a single cell, but randomly aligned to the myonemes in surrounding cells (Seybold et al., 2016). Only at about 48 hpd the myonemes of each cell align to form a coordinated muscle system. The polarization process takes place in a very comparable manner within the ectodermal and the endodermal epithelia, though the two muscle networks will ultimately be orthogonally oriented (Seybold et al., 2016). Further work is required to characterize the molecular mechanisms underlying not only the cellular autonomous repolarization of the myonemes in *Hydra*, but also how the individual cells communicate in order to form the polarized and coordinated muscle networks.

### Striated muscle transdifferentiation in *Podocoryna*

Cellular plasticity plays a crucial role in most regenerative processes. The most extensive work aimed at understanding muscle plasticity in cnidarians, was carried out by Schmid and colleagues in the hydrozoan jellyfish *Podocoryna carnea*. In a seminal series of papers they detailed the remarkable transdifferentiation process able to convert isolated striated muscle cells to neuronal or smooth muscle fates (Figure 6E) and to ultimately regenerate a fully functional manubrium (reviewed in Schmid, 1988; Schmid et al., 1988; Brockes, 1994; Reber-Müller et al., 1995).

From the subumbrella of the medusa *Podocoryna carnea*, endodermal and striated muscle layers can be isolated and cultivated for weeks. Following collagenase treatment to disrupt muscle and ECM interaction, striated muscle cells transdifferentiate into smooth-like muscle cells (Schmid, 1978, 1992). They lose their striated myofibrils, develop a cilium and adopt a morphology that is similar to smooth muscle cells. This process is transcription and translation but not proliferation dependent (Schmid, 1975; Weber et al., 1987). Once transdifferentiated, those cells behave in a stem cell fashion, as they self-renew and give rise to a differentiated cell, following a strict pattern (Figure 6E). In fact, each subsequent division results in a nerve cell expressing the neurotransmitter FMRF-amide and a cycling smooth muscle cell (Alder and Schmid, 1987).

Combined with isolated endodermal cells of the umbrella, isolated striated muscle cells can regenerate a functional manubrium containing at least seven new cell types, including gametes (Schmid, 1974, 1976; Schmid et al., 1982). Further refinement of the cell separation protocol allowed Schmid and colleagues to obtain a fully regenerated manubrium from a pure population of destabilized (collagenase treated) striated muscle cells (Schmid and Alder, 1984). These transdifferentiation experiments were also successfully performed using striated muscles of other hydrozoan medusae (Schmid, 1978) albeit with lower efficiency than in *Podocoryna carnea*.

A number of genes expressed in *Podocoryna* striated muscle cells and whose expression is altered during transdifferentiation, have been characterized. While *twist* is likely not involved in this process (Spring et al., 2000), *msx* expression is downregulated in response to cellular dissociation and strongly reactivated during smooth-like muscle differentiation (Galle et al., 2005). *bmp2/4* expression is initiated immediately after excision and *bmp5/8* in the initial phase of the transdifferentiation process (Reber-Müller et al., 2006). Interestingly, expression of the *Podocoryna* Piwi homolog, *cniwi*, is upregulated during transdifferentiation (Seipel et al., 2004) and potentially involved in the potency (Van Wolfswinkel, 2014) of the muscle cells to become neurons. Although these data suggest an implication of the transcription factor Msx, the RNA-binding protein Cniwi and BMP signaling in the transformation potential of striated muscles in *Podocoryna*, no functional data is available.

### Muscle plasticity during jellyfish self-repair

Following up on this *in vitro* work on *Podocoryna*, Lin and colleagues analyzed wound healing and remodeling of the striated muscle cells *in toto*, in the umbrella of the jellyfish *Polyorchis penicillatus* (Lin et al., 2000). After wounding, the striated muscles cells can lose their condensed actin fibers and dedifferentiate, enabling them to migrate toward the wound. During the migration process, which is dependent on intracellular calcium resources, the cells lose also their contractile ability while surrounding intact epitheliomuscular cells remain able to contract in response to a chemical stimulus (Lin et al., 2000). The dedifferentiation-migration response to wounding takes about 8–10 h and does not seem to involve cell proliferation. Once the dedifferentiated cells have filled up the wound site, they stop migrating and begin to re-differentiate and re-polarize, for finally becoming fully functional muscle cells within 24–48 h post injury (Lin et al., 2000).

In culture, the migrating striated muscle cells in *Podocoryna* induce a change in gene expression that is rapidly communicated to the non-migrating cells, thus allowing a coordinated tissue reorganization (Yanze et al., 1999). In addition to the transdifferentiation potential of striated muscle cells *in vitro*, these observations show the de- and re-differentiation capacity of the same muscle cell type *in vivo*.

Another hydrozoan jellyfish that has been used to understand medusa self-repair mechanism is *Clytia hemisphaerica* (previously named *Phialidium hemisphaericum* or *Campanularia johnstoni*). Differently sized fragments of the jellyfish umbrella are able to rapidly restore the bell shape by a “morphodynamic process” and subsequently reform, at various degrees, missing structures such as the canals, tentacles and gonads (Figure 6B; Schmid and Tardent, 1971; Schmid et al., 1976). The mechanisms by which *Clytia* medusae reform the missing structures and restore the bell shape are still unknown.

More recently, the scyphozoan *Aurelia aurita* was used to gain insights into a particular mechanism that enables injured ephrya (juvenile jellyfish) to rapidly regain a functional shape and pursue its development into adulthood (Abrams et al., 2015). In this specific case, the “healing” process does not involve cellular proliferation or apoptosis but a so-called symmetrization (term introduced by Abrams et al., 2015). This process reshapes the animal by reorganizing the existing parts, without reformation of the missing parts (Figure 6C). This self-repairing event is crucial to allow subsequent development of the damaged juvenile jellyfish into a radially symmetrical adult. Unlike during regeneration in other cnidarians (Chera et al., 2009; Passamaneck and Martindale, 2012; Amiel et al., 2015), inhibition of cellular proliferation or apoptosis does not affect the symmetrization process in *Aurelia aurita* (Abrams et al., 2015). Interestingly, inhibiting the muscle reconnection following injury using low doses of cytochalasin D (to avoid nonspecific actin dependent effects and to still allow contraction of the existing muscles) does not affect symmetrization (Abrams et al., 2015). However, further analyses using muscle relaxants, such as magnesium chloride or menthol causing the decrease of the pulsation frequency and the inhibition of symmetrization, revealed that contraction forces that are generated by the musculature network of the juvenile jellyfish are likely important for this process (Abrams et al., 2015). If this contraction dependent symmetrization process is specific to *Aurelia aurita* ephrya, or represents a general strategy for maintaining the swimming capacity in injured adult jellyfish, is currently unknown.

### Muscle and regeneration in *Nematostella*

*Nematostella vectensis* is emerging as a new regeneration model (Reitzel et al., 2007; Trevino et al., 2011; Passamaneck and Martindale, 2012; Bossert et al., 2013; DuBuc et al., 2014; Amiel et al., 2015), particularly well-suited to compare development and regeneration within the same organism (Burton and Finnerty, 2009; Layden et al., 2016). Recent studies have analyzed its basic regeneration capacity (Figure 6D; Reitzel et al., 2007; Amiel et al., 2015), establishing precise staging systems to analyze the regeneration process under physiological and perturbation conditions (Bossert et al., 2013; Amiel et al., 2015), and developing new *in vivo* tools to asses wound healing, pharynx formation and tissue tracing (Amiel et al., 2015). These studies have shown that cellular proliferation is induced at the amputation site and required for the regeneration process (Passamaneck and Martindale, 2012; Amiel et al., 2015). Additional work in *Nematostella* is required to identify stem and progenitor cells.

The muscle regeneration process in *Nematostella* was initially studied with a MyHC1::mCherry transgenic line labeling the retractor muscles (Renfer et al., 2010). Immediately after amputation, these muscles retracted from the wound site. Later in the process, numerous cells accumulated at the regenerating site expressing the MyHC1::mCherry transgene (in a non-polarized manner) suggesting active cellular differentiation and reorganization events in this region (Renfer et al., 2010). However, nothing is known about the cellular origin of the newly formed retractor muscle fibers, nor the cellular and molecular mechanisms underlying this process.

A recent study suggested that muscle contraction could play a role in regeneration of missing body parts (Bossert et al., 2013). In fact, contraction of the circumferential muscle fibers may be involved in reducing the size of the wound in isolated adult physa (the most aboral part of the polyp in burrowing actiniaria) and thus, potentially promoting the wound healing process and the reformation of oral structures. A detailed characterization of the oral regeneration process in juveniles shows a very stereotyped and dynamic behavior of the tissues during the regeneration program (Amiel et al., 2015), suggesting that muscle contractions may play a role also during later steps of regeneration. Additional analyses are required to understand the process of muscle fiber regeneration and repolarization as well as the role that muscles and muscle contractions play during wound healing and regeneration in *Nematostella*.

## Conclusion and perspectives

In this overview, we have introduced the cnidarians (Figure 1), a group of animals with diverse life cycles (Figure 2) and holding a key phylogenetic position as the sister group to bilaterians. Cnidarian muscles are composed of a set of epitheliomuscular cell types and, in some species, include additional, independently–evolved, striated muscles (Figure 4) and myocytes. The epitheliomuscular cells play a role in prey capture, locomotion or defense from predators (Figure 3). Intriguingly, cnidarians possess genes that are generally associated with muscle formation in bilaterians (e.g., Mef2), but lack classical myogenic regulatory factors such as MyoD (Figure 5) as well as terminal differentiation proteins typical of bilaterian striated muscles such as the Troponins and Titin. Cnidarians possess quite extraordinary regenerative capacities as they can regrow missing body parts from isolated fragments or in some species even from dissociated cells aggregations (Figure 6). Although the regenerative capacity has intrigued scientists for over 300 years, currently little is known about their capacity to reform/regenerate injured muscles. In order to better understand the similarities and differences of muscle plasticity in cnidarians, an emphasis has to be put on carrying out functional studies in existing as well as new models. Recent technological advances will be greatly beneficial for both aspects.

### Open questions in cnidarian muscle development and regeneration

As described above, muscle plasticity has only been studied in a handful of cnidarians and is to date rather descriptive. Work carried out in jellyfish suggest that de- and re-differentiation as well as cell migration might be involved in wound healing and reformation of striated muscle fiber network. The fact that cellular proliferation is not detected during this process raises the question about how cellular homeostasis is maintained. Are there undifferentiated precursors involved in the wound healing process as well? Which are the molecular signals inducing dedifferentiation process, cellular migration and its guidance, as well as the re-differentiation into striated muscles? While latter questions are rather medusa specific, there are also questions that are relevant to all cnidarians. How are the condensation and the polarization of the actin fibers controlled to reform a perfectly organized and integrated muscle network? What are the molecular cues responsible for myoneme polarization during re-aggregation/regeneration experiments in *Hydra*? What controls the suggested de- and re-differentiation of retractor muscle cells during oral regeneration in *Nematostella*? Does this anthozoan possess epithelial stem cells similar to the ones described in hydrozoans or do they possess multi-potent stem cells? What causes the condensation and orientation of the thick longitudinal retractor muscle fibers, compared to the thin circumferential muscle fibers, both residing in the endodermal epithelium? Are those dependent on signaling molecules released by the mesenteries, mechanical forces induced by mesenterial infoldings, or a combination of the two? Do “smooth-muscle-like” epitheliomuscular cells possess transdifferentiation potential similar to the ones described from striated jellyfish muscle cells? Answering this non-exhaustive catalog of open questions is not only important to provide new insights into cnidarian muscle plasticity, but will also help providing a better understanding of the mechanisms underlying initial cnidarian muscle development.

### Understanding muscle polarization in cnidarians

Epitheliomuscular cells associate to form condensed muscle fibers (e.g., muscle net in *Hydra* Figures 4Aa,a' or longitudinal muscle fibers in *Nematostella*, Figures 4Cc,c') in diverse orientations (Figures 4A–C). While recent work has nicely described the repolarization process in *Hydra* (Seybold et al., 2016), the molecular and/or mechanical signals that control the condensation and orientation/polarization of cnidarian muscle fibers during development are unknown. A study using transgenic *Nematostella* MyHC1::mCherry (Renfer et al., 2010) has shown that condensed muscle fibers of the retractor muscles appear progressively during the late planula-primary polyp transition (Jahnel et al., 2014). One intriguing aspect of muscle development in *Nematostella* is that the polarity of epitheliomuscular cells within the same endodermal epithelium varies according to their spatial coordinates. The myonemes localized in the portions of the body column in-between the mesenteries form the circumferential ring musculature, while those included at and in the mesenteries (parietal and retractor muscles) are oriented longitudinally (Figures 4Cc,c'; Jahnel et al., 2014). As the infolding of the endodermal epithelium is likely contributing to the formation of the mesenteries (Jahnel et al., 2014; Leclère and Rentzsch, 2014), it would be interesting to investigate the mechanical aspects of this process, by looking at the role that mechanical forces play on the orientation of the myonemes, or conversely, the role that longitudinal muscle fibers have on the guidance/formation of mesenteries.

### Cnidarians as new models to study myoepithelial development/plasticity

Studies on cnidarians (Figure 1) could help gaining insights into the evolution of the mesodermal germ layer (absent in cnidarians, but present in bilaterians) and thus about those tissues that in bilaterians are mesodermal derivatives, such as muscles (reviewed by Seipel and Schmid, 2006; Burton, 2008; Technau and Steele, 2011; Layden et al., 2016). The developmental program of cnidarian muscles is currently largely unknown and requires intense functional work. Thus, addressing this question has been initiated by studying the expression (Spring et al., 2000, 2002; Fritzenwanker et al., 2004; Martindale et al., 2004) and function (Genikhovich and Technau, 2011) of “mesodermal” genes (e.g., *brachyury, mef2*) or the gene regulatory networks controlling endomesoderm development (Röttinger et al., 2012). However, these studies have an undeniable bias, the implicit assumption being that cnidarian muscle cells are essentially similar to bilaterian muscles. If on one hand the hypothesis of an independent origin for cnidarian and bilaterian striated muscles has been taken into account (Steinmetz et al., 2012), on the other hand all other cnidarian muscle cell types have generically been considered as “smooth muscle cells” (Seipel and Schmid, 2005; Burton, 2008; Steinmetz et al., 2012). The latter statement is supported by the fact that they are mononucleated and express “smooth muscle” proteins such as Myosin Heavy Chain (Renfer et al., 2010).

It is however important to keep in mind that the embryological origin of cnidarian muscle cells is not the mesodermal germ layer, but either the endodermal or the ectodermal layer and, importantly, that cnidarian muscle cells are mostly epithelial. As for cnidarian epitheliomuscular cells, bilaterians myoepithelial cells originate from tissues of various developmental origins (Petersen and van Deurs, 1989; Schmidt-Rhaesa, 2007; Tamgadge et al., 2013). Furthermore, myoepithelial cells in mammalians are receiving increasing interest, because of their importance in processes such as gland development, growth and differentiation, in pathologies such as breast cancer (Silva et al., 2015) as well as their capacity to control tumorigenesis (Gudjonsson et al., 2005; reviewed in Deugnier et al., 2002; Sopel, 2010). Thus, it could be particularly fruitful to compare bilaterian myoepithelial and cnidarian epitheliomuscular cells, their developmental origin, the molecular or mechanical signals that control their polarization, condensation and organization into muscle nets, rings or fibers and how they regenerate after injury. The easy access to biological material offered by cnidarian models, combined with various modern approaches that are now routinely performed on these organisms make them very interesting models to investigate myoepithelium formation and regeneration.

### Potential roles of muscles in cnidarian regeneration

Recent work suggests that muscle contraction could play a primary role during the regenerative process, by promoting wound healing in *Nematostella* (Bossert et al., 2013) and allowing juvenile scyphozoan jellyfish to reshape rapidly into a functional body, in a process recently called “symmetrization” (Abrams et al., 2015). Interestingly, mammary myoepithelial cells, in addition to their contractile function, preserve also the regenerative potential of the tissue and are able to modulate in an integrin mediated process the proliferation and differentiation of surrounding cells (Deugnier et al., 2006; Shackleton et al., 2006; Stingl et al., 2006; Sleeman et al., 2007; reviewed in Moumen et al., 2011). In planarians, which similarly to cnidarians display impressive regenerative capacities, several lines of evidence show that the vast majority of “position control genes,” (Reddien, 2011) such as *wnt1* or *notum* (Adell et al., 2009; Petersen and Reddien, 2011), are not only responsible for the polarity and patterning events during regeneration, but are also expressed in the muscles cells (Witchley et al., 2013). These observations led to the hypothesis that planarian muscle cells provide positional information to the surrounding stem cells, thus promoting regional differentiation and body polarization (Witchley et al., 2013; reviewed in Cebrià, 2016). Along these lines, it would be particularly interesting to address whether cnidarian epitheliomuscular cells and/or muscle networks play a role during wound healing and subsequent regenerative processes by providing biochemical or biomechanical cues.

The development of new molecular tools in a handful of hydrozoan and anthozoan species has already provided new insights into several long-standing evolutionary and developmental questions (Houliston et al., 2010; Technau and Steele, 2011; Galliot, 2012; Nebel and Bosch, 2012; Plickert et al., 2012; Sinigaglia et al., 2013; Layden et al., 2016; Rentzsch and Technau, 2016). Those tools, in combination with “omics” and functional genomics approaches (Momose and Houliston, 2007; Rentzsch et al., 2008; Amiel et al., 2009; Chera et al., 2009; Genikhovich and Technau, 2009; Boehm et al., 2012; Röttinger et al., 2012; Layden et al., 2013; Lapébie et al., 2014; Bradshaw et al., 2015) as well as with the recently developed techniques for genome editing (Ikmi et al., 2014) are now opening new opportunities to functionally and thoroughly address the developmental and regenerative program of cnidarian muscles systems, but also the role(s) that epitheliomuscular cells, muscle fibers and muscle contraction can play on the regeneration process.

## Author contributions

All authors listed, have made substantial, direct and intellectual contribution to the work, and approved it for publication.

### Conflict of interest statement

The authors declare that the research was conducted in the absence of any commercial or financial relationships that could be construed as a potential conflict of interest.
